# Impact of humanised isolation and culture conditions on stemness and osteogenic potential of bone marrow derived mesenchymal stromal cells

**DOI:** 10.1038/s41598-019-52442-9

**Published:** 2019-11-05

**Authors:** Salwa Suliman, Hassan R. W. Ali, Tommy A. Karlsen, Jerome Amiaud, Samih Mohamed-Ahmed, Pierre Layrolle, Daniela E. Costea, Jan E. Brinchmann, Kamal Mustafa

**Affiliations:** 1 Department of Clinical Dentistry, Centre for Clinical Dental Research, University of Bergen, Bergen, Norway; 2 Norwegian Center for Stem Cell Research, Department of Immunology, Oslo University Hospital Rikshospitalet, Oslo, Norway; 3 INSERM, UMR 1238, PHY-OS, Laboratory of Bone Sarcomas and Remodeling of Calcified Tissues, Faculty of Medicine, University of Nantes, Nantes, France; 4 Gade Laboratory for Pathology, Department of Clinical Medicine, University of Bergen, Bergen, Norway; 5 Department of Pathology, Haukeland University Hospital, Bergen, Norway; 6 Centre for Cancer Biomarkers, University of Bergen, Bergen, Norway; 7 Department of Molecular Medicine, Faculty of Medicine, University of Oslo, Oslo, Norway

**Keywords:** Mesenchymal stem cells, Translational research, Stem-cell research

## Abstract

Therapeutic potential of human bone marrow stromal/stem cells (hBMSC) must be developed using well defined xenogenic-free conditions. hBMSC were isolated from healthy donors (n = 3) using different isolation and expansion methods. Donor I was isolated and expanded by either bone marrow directly seeded and cells expanded in 10% AB human serum (AB) +5 ng/ml fibroblast growth factor-2 (FGF2) [Direct(AB + FGF_low_)] or Ammonium-Chloride-Potassium Lysing Buffer was used before the cells were expanded in 10% AB +5 ng/ml FGF-2 [ACK(AB + FGF_low_)] or Lymphoprep density gradient medium was used before the cells were expanded in 10% AB +5 ng/ml FGF2 [Lympho(AB + FGF_low)_] or bone marrow directly seeded and cells expanded in 10% pooled platelet lysate plasma (PL) + heparin (2 I/U/mL) [Direct(PL)]. Groups for donors II and III were: Direct(AB + FGF_low_) or 10% AB +10 ng/ml FGF2 [Direct(AB + FGF_high_)] or Direct(PL). HBMSCs were assessed for viability, multi-potency, osteogenic, inflammatory response and replicative senescence *in vitro* after 1 and 3 weeks. Pre-selected culture conditions, Direct(AB + FGF_high_) or Direct(PL), were seeded on biphasic calcium phosphate granules and subcutaneously implanted in NOD/SCID mice. After 1 and 11 weeks, explants were analysed for inflammatory and osteogenic response at gene level and histologically. To identify implanted human cells, *in situ* hybridisation was performed. hBMSC from all conditions showed *in vitro* multi-lineage potency. hBMSCs expanded in PL expressed stemness markers *in vitro* at significantly higher levels. Generally, cells expanded in AB + FGF2 conditions expressed higher osteogenic markers after 1 week both *in vitro* and *in vivo*. After 11 weeks *in vivo*, Direct(AB + FGF_high_) formed mature ectopic bone, compared to immature mineralised tissues formed by Direct(PL) implants. Mouse responses showed a significant upregulation of IL-1α and IL-1β expression in Direct(PL). After 1 week, human cells were observed in both groups and after 11 weeks in Direct(AB + FGF_high_) only. To conclude, results showed a significant effect of the isolation methods and demonstrated a relatively consistent pattern of efficacy from all donors. A tendency of hBMSC expanded in PL to retain a more stem-like phenotype elucidates their delayed differentiation and different inflammatory expressions.

## Introduction

Mesenchymal stromal/stem cells are progressively being used in all arenas of tissue engineering and cell-based therapies^1,2^. With regard to bone regeneration, human bone-marrow derived mesenchymal stromal/stem cells (hBMSC) present advantages over other sources of MSC^3^ and over pluripotent cell types such as induced pluripotent stem cells due to their autologous mode of use, which require less extensive *in vitro* manipulation or ethical clearance, associated with a lower risk^4^. hBMSC are rare cells, population ranges from 0.001% to 0.01% of the total number of nucleated cells present in bone marrow^5^. Pertaining to this drawback, *in vitro* cell expansion in monolayers is the most commonly used approach to produce sufficient cell numbers prior to pre-clinical or clinical implantations. Despite the increasing number of clinical trials, culturing conditions for hBMSC are still under development^6^. There is substantial evidence that the *in vitro* expansion phase affects their phenotype, with considerable implications for the development of effective therapies. With hBMSC-based therapies overtaking clinical applications in bone regeneration and establishing a new clinical paradigm^1,2^, the development of production methods in accordance with current Good Manufacturing Practices (GMP) is mandatory for a safe and efficient regeneration^6,7^. In compliance with the European Commission regulation 1394/2007, hBMSC are considered advanced therapy medicinal products in Europe^8^.

Clinical translation trials in accordance with GMP require the use of a well-defined culture medium when expanding hBMSC to avoid adverse reactions in patients^6^. Foetal bovine serum (FBS) is derived from the whole blood of bovine foetuses and it is a rich source of essential growth factors. These include platelet derived growth factor (PDGF), transforming growth factor beta 1 (TGFβ-1), fibroblast growth factor 2 (FGF2), vascular endothelial growth factor (VEGF), insulin-like growth factor (IGF), growth hormones and albumin, making it the optimum and most broadly used supplement for expansion of hBMSC^9^. However, it comes with safety concerns such as zoonotic infections since it contains enogeneic antigens as well as ethical concerns^9,10^. In addition, the concentrations of growth factors in FBS are difficult to control between production batches, and even clinical-grade FBS is reported to show variability between its inherent composite of bioactive factors^9^. To address these issues, alternative animal-free strategies are currently being developed for the provision of nutrients and attachment factors for culture and expansion of hBMSC. These are generally divided into chemically defined media, and ‘humanised’ supplements derived from human blood derivatives. The proposed derivatives include: autologous or allogeneic human serum, human platelet derivatives, cord blood serum and human plasma derivatives^11^.

When comparing hBMSC expanded using human serum to those cultured using FBS, promoted proliferation and enhanced gene expressions with genomic stability were portrayed^12^. Studies mainly using autologous serum revealed potential for expansion and osteogenic differentiation of hBMSC; however this potency was shown to be age dependant^13^. Reports on allogeneic serum have been contradictory, and pooling of blood samples seems to reduce variability^12,14^. Use of autologous serum presents with limitations, for instance availability of large quantities required for clinical applications^15^. Therefore, alternatives such as pooled human serum from type AB donors were introduced.

The physiological role of blood platelets in tissue repair justifies the use of their derivatives in regeneration. Human platelet lysate (PL) can be obtained from platelets using different procedures (*e.g*. thrombin activation, freeze/thaw platelet lysis or sonication). It contains an extensive variety of growth factors and cytokines such as VEGF, FGF2, epidermal growth factor (EGF), IGF, PDGF and TGF-β1 in higher levels relative to human serum^16^. PL has shown superior efficacy when compared to FBS-propagated cultures. hBMSC were shown to be more responsive to chondrogenic and adipogenic stimulation when cultured in PL compared to FBS^17^. Among the advantages of PL is the constancy of its bioactivity even when using expired platelet concentrates^18^, proving comparable proliferation and osteogenic potential when used to culture hBMSC^19^. PL is highly dependent on the preparation method, donor age and blood profile, which makes it difficult to accurately characterise its constituents; however, pooling can reduce variances^20^. To avoid the fibrin clots from forming when human PL is added to the culture medium, which contains calcium, heparin is usually added to human PL. The levels of heparin have shown to variably interfere with the growth rate of cells^18^. Furthermore, concerns of immunological responses and transmission of human infections are also valid as for other human-derived media supplements.

Variation in expansion conditions influences the efficacy and differentiation potential of hBMSCs. This is an important consideration when designing tissue engineering and regenerative studies. Most reports comparing the human alternatives to FBS have provided contradictory results in their proliferation and differentiation potencies. Only few reports have done an inter-comparison among the different human alternatives to highlight the actual differences in efficacy^21^. Increasing cell yield, while maintaining stemness, represents a significant challenge for the *in vitro* expansion of clinical grade hBMSC. Recently, we reported a Phase 1 clinical trial to regenerate dentoalveolar bone defects where autologous hBMSC were expanded in GMP-grade PL from human pooled platelet concentrates as growth factor supplement^22^. In attempts to improve these protocols and transfer technologies, the current study compares different isolation methods of hBMSC and further expansion in different human-derived culture media, namely, human AB serum (AB) supplemented with FGF2 or PL. To evaluate the regenerative therapeutic capacity of these cells expanded using different isolation and culture conditions, a systematic assessment was carried out both *in vitro* and *in vivo* in an ectopic rodent model.

## Methods

### Pooled human platelet lysate preparation

PL plasma was prepared according to published protocols^23^ with minor modifications. Briefly, pooled platelets from 4 donors suspended in platelet additive solution was spun at 1700 g at room temperature (RT). The resulting pellet was re-suspended in 10 mL Octaplas AB plasma (Octapharma AS, Jessheim, Norway) and frozen at −20 °C. This constituted one unit. After thawing, platelets from 19 units were pooled and adjusted to a final volume of 4.8 L with Octaplas AB, and subjected to a second freeze-thaw-cycle before being centrifuged at 4000 g at 4 °C for 15 min to remove platelet cell wall fragments, and subsequently frozen in aliquots. When PL was added to culture medium, 2I/U per mL heparin was added, following previously optimised protocols^20^, to avoid coagulation of the medium through clumping of the fibrinogen in the plasma. The platelets used to make PL are all originally donated for use for patients. However, if they are not used after 7 days of storage they are deemed unfit for use in patients, and will normally be destroyed, or used for research. We have tested, and found that they may still be used to make PL, as the alternative is that these platelets are destroyed. The donors are anonymous to the research laboratory, and the platelet or platelet lysate is not being subject to any type of analysis. Ethical approval to use PL in culture conditions was obtained from the Regional Committees for Medical and Health Research Ethics in Norway (REK approval no. 2016/1266). All procedures were performed in accordance with the relevant guidelines and regulations in the blood bank.

### Human bone marrow stromal/stem cells isolation and expansion

HBMSC were isolated from the bone marrow of three different healthy donors. A total of 30 to 60 mL of bone marrow was obtained by aspirations from the iliac crest under local anaesthesia, after ethical approvals were obtained from the Regional Committees for Medical and Health Research Ethics in Norway (REK approval no. S-07043a). All donors gave a written, informed consent and all procedures were performed in accordance with the relevant guidelines and regulations. The donors were 28, 37 and 37 years of age. The cells were characterised and assessed after isolation using different bone marrow processing conditions and expansion culture conditions. Briefly, the whole bone marrow from the first donor was divided equally and the different isolation and expansion conditions used are summarised in Table 1.

**Table 1:** Description of the different isolation and expansion conditions for Donor I.

DONOR I	
Abbreviation	Description
Direct(AB + FGF_low_)	Bone marrow was directly seeded onto flasks with α-MEM +10% AB serum +1% P/S and 5 ng/mL FGF2 (all from R&D)
ACK(AB + FGF_low_)	Prepared with Ammonium-Chloride-Potassium (ACK) lysing buffer (Gibco) and cells were seeded with α-MEM +10% AB serum +1% P/S and 5 ng/mL FGF-2
Lympho(AB + FGF_low)_	Bone marrow was centrifuged in a density gradient medium Lymphoprep (Stemcell Technologies) to isolate mononuclear cells before culturing in α-MEM +10% AB serum +1% P/S and 5 ng/mL FGF2 (all from R&D)
Direct(PL)	Bone marrow was directly seeded onto flasks with α-MEM +1% P/S (Gibco) +10% PL + heparin (2I/U per mL)

Abbreviations: α-MEM, alpha minimum essential medium; AB serum, human serum from type AB donors; P/S, penicillin/streptomycin, FGF2, fibroblast growth factor 2.

All methods involving humans were performed in accordance with the relevant guidelines and regulations.

The bone marrow from the 2^nd^ and 3^rd^ donors was equally divided and directly seeded without prior processing and expansion conditions are described in Table 2.

**Table 2:** Description of the different isolation and expansion conditions for Donor I and III.

DONORS II and III	
Abbreviation	Description
Direct(AB + FGF_low_)	Bone marrow was directly seeded onto flasks with α-MEM +10% AB serum +1% P/S and 5 ng/mL FGF2
Direct(AB + FGF_high_)	Bone marrow was directly seeded onto flasks with α-MEM +10% AB serum +1% P/S and 10 ng/mL FGF2
Direct(PL)	Bone marrow was directly seeded onto flasks with α-MEM +10% AB serum +1% P/S +10% PL + heparin (2I/U per mL)

Abbreviations: α-MEM, alpha minimum essential medium; AB serum, human serum from type AB donors; P/S, penicillin/streptomycin, FGF2, basic fibroblast growth factor 2.

The cells were expanded for 2 passages at a laboratory at Norwegian Center for Stem Cell Research in Oslo University Hospital before they were transferred to the laboratory at University of Bergen for further *in vitro* differentiation and *in vivo* osteogenic potential evaluation. The viability of the cells before and after shipment was assessed using trypan blue dye.

### Multipotent capacity *in vitro*

Cells were seeded (3–5 × 10^3^ per cm^2^) in 12-well plates (NUNC, Thermo Fisher Scientific, Waltham, USA). Differentiation was induced 2–5 days from seeding (depending on the cells’ confluency) using adipogenic medium for 2 weeks and osteogenic media for 3 weeks (all from StemPro, Thermo Fischer Scientific). For detection of adipogenic differentiation, cells were stained with Oil Red O stain, while mineralisation was detected by Alizarin red staining as described previously^24^.

### Multipotency, inflammatory response, osteogenic and adipogenic differentiation potential and replicative senescence *in vitro* at gene level

Using a Tissue RNA isolation kit (Maxwell, Promega, Madison, WI, USA), total RNA was isolated from *in vitro* osteogenically differentiated hBMSC cultures after 1 and 3 weeks and undifferentiated controls at 1 week only. Quantity and purity were checked using a Nanodrop spectrophotometer (Thermo Fisher Scientific). Total RNA was reverse transcribed according to manufacturer’s instructions using the High-capacity complementary DNA Reverse Transcription Kit (Applied Biosystems, Carlsbad, CA, USA). Quantitative real time PCR was conducted on a StepOne Plus system (Applied Biosystems), using TaqMan gene expression assays (Applied Biosystems). Gene expression assays (Taqman) (Applied Biosystems) were used to detect mRNA levels of glyceraldehyde-3-phosphate dehydrogenase (GAPDH), octamer-binding transcription factor 4 (Oct-4), NANOG and Cluster of Differentiation 90 (CD90) and the inflammatory markers interleukin 1 alpha (IL-1α), beta (IL-1β), interleukin 6 (IL-6) and 8 (IL-8) in the undifferentiated controls. mRNA levels of runt-related transcription factor 2 (RUNX2), collagen type 1 alpha 1 (Col 1α1), alkaline phosphatase (ALP), bone morphogenetic protein-2 (BMP-2), bone sialoprotein (BSP), osteocalcin (OC) and GAPDH were detected from osteogenically differentiated cultures at 1 and 3 weeks. mRNA levels of adipogenic markers, peroxisome proliferator-activated receptor gamma (PPARG), CCAT/enhancer-binding protein alpha (CEBPA) and lipoprotein lipase (LPL) were detected from adipogenically differentiated cultures at 2 weeks. To evaluate replicative senescence, mRNA level of upregulated genes during cellular aging, Rho GTPase activating protein 29 (PARG1/ARHGAP2) and cyclin dependent kinase inhibitor 2 A (CDKN2A) and downregulated gene pleiotrophin (PTN) were evaluated from osteogenically differentiated cells at 1 and 3 weeks. The data were analysed with a comparative C_T_ method and GAPDH served as an endogenous control. Primer details are summarised in Supplementary Table 1.

### Surface markers evaluation

The results obtained from the *in vitro* characterisation determined the direct seeding isolation method for further evaluation of osteogenic efficacy *in vivo*. Before seeding on scaffolds, the cells from Direct(AB + FGF_high_) or Direct(PL) were stained with mouse anti-human CD44-FITC (Bio SB), CD90-PE (eBioscience), HLA-A,B,C-APC (BD Biosciences, USA), HLA-DR-APC (eBioscience), CD105-FITC (Sigma), CD14-FITC (Sigma), CD73-PE (BD Biosciences), CD34-FITC (BD Biosciences), CD45-FITC (eBioscience) for 15 min at RT, before being washed and re-suspended in PBS. Mouse anti-human immunoglobulin isotype antibodies (eBioscience) were used as controls. Acquisition was performed using a BD LSRFortessa cell analyser (BD Biosciences) and data were analysed using FlowJo (V10, Flowjo LLC, Ashland, OR, USA).

### Cell/scaffold constructs’ preparation and subcutaneous implantation

HBMSC from Direct(AB + FGF_high_) or Direct(PL) without being osteogenically differentiated were seeded on biphasic calcium phosphate (BCP) granules (MBCP; Biomatlante, France) ranging in size from 1–2 mm. Cells (7.5 × 10^3^) per mg were seeded on 65 mg of BCP placed in a 96-well plate. Cell-free BCP (BCP alone) served as controls. The plates were then shaken in a plate shaker to allow distribution of the cells within the granules and incubated overnight at 37 °C and 5% CO_2_. Two, 1 cm incisions, were made on the back of 8–10 weeks old non-obese diabetic/severe combined immunodeficiency (NOD/SCID) mice. The two groups of cell/scaffold constructs or the BCP alone groups were randomly distributed and each animal got a total of four subcutaneous implants (n = 8 for each group). The mice were sacrificed after 1 and 11 weeks. All animal experiments were approved by the Norwegian Animal Research Authority and conducted in strict accordance with the European Convention for the Protection of Vertebrates used for Scientific Purposes (FOTS no. 7894).

### Osteogenic differentiation potential and inflammatory response *in vivo* at gene level

Total RNA was isolated from *in vivo* scaffolds 1 week post-implantation and PCR was performed as described previously for the *in vitro* samples. Taqman gene expression assays (Applied Biosystems, USA) were used to detect mRNA levels of GAPDH, RUNX2, Col1α2, ALP, IL-1α, IL-1β, IL-6 and IL-8 for human and mice. The data were analysed with a comparative CT method, with GAPDH as an endogenous control and BCP alone as the reference group.

### Histological analysis and histomorphometry

After fixation in 4% paraformaldehyde, the harvested *in vivo* scaffold constructs were decalcified in EDTA and cut into half before being processed for paraffin embedding. Sections (3–4 µm) were stained with haematoxylin and eosin (H&E). The histological sections were scanned using an Aperio Scanscope Scanner (Aperio Vista, CA, USA) and viewed through Aperio ImageScope software program. Qualitative and semi-quantitative histological evaluations were carried out to assess the amount of bone formed. The frequency of bone formation was estimated as the number of scaffolds with newly formed bone related to the total number of implanted scaffolds.

### *In-situ* hybridisation of the human Alu-sequence

To identify the hBMSC in the cell/scaffold constructs harvested after the *in vivo* implantation, *in situ* hybridisation using the human-specific repetitive Alu sequence was performed as previously described^25^. Briefly, paraffin sections were treated for heat induced epitope retrieval for 20 h at 60 °C in citrate buffer 10 mM (pH 6) plus Tween 20 0.05% followed by 0.25% acetic acid in 0.1 M triethanolamine (pH 8) for 20 minutes at RT. Prehybridisation was performed for 3 hours at 56 °C in a hybridisation buffer containing 4 × Saline-Sodium Citrate buffer (SSC) (Sigma-Aldrich), 50% deionised formamide, 1 × Denhardt’s solution, 5% dextran sulfate, 100 μg/ml salmon sperm DNA and molecular-grade water. Hybridisation buffer was refreshed with the addition of 70 nM custom DIG-labelled human locked nucleic acid Alu probe 5DigN/5′-TCTCGATCTCCTGACCTCATGA-3′/3DigN (Exiqon, Vedbaek, Denmark) and then target DNA and the probe were denatured for 1 h  at 70 °C. Hybridisation was carried out overnight at 56 °C. The hybridised probe was detected by immunohistochemistry using biotin-SP conjugated IgG fraction monoclonal mouse anti-digoxin (Jackson Immunoresearch, West Grove, Pennsylvania, USA) diluted 1/400 in Tris-buffered saline with Tween, 2% bovine serum albumin for 1 h at RT. Stretoperoxidase was added (1/400 in Tris-buffered saline with Tween) for 35 min at RT. All bound reactions were visualised with 3,3′ diaminobenzidine substrate (Dako, Les Ulis, Ile-de-France, France). Sections were counterstained with Gill-2 hematoxylin (Thermo Shandon Ltd, Runcorn, UK).

### Statistical analyses

All data are presented as the mean values + standard error of the mean. Average values were analysed using SPSS Statistics 25.0 (IBM, Armonk, NY, USA). Data were tested for variance homogeneity and normal distribution and One-way ANOVA were followed by a multiple- comparison LSD test. Differences between the means were considered statistically significant when p < 0.05.

### Ethics approval

Bone marrow samples were collected after ethical approvals were obtained from the Regional Committees for Medical and Health Research Ethics in Norway (REK Approval No. S-07043a). The samples were collected following an informed consent of the patients. All animal experiments were approved by the Norwegian Animal Research Authority and conducted in strict accordance with the European Convention for the Protection of Vertebrates used for Scientific Purposes (FOTS No. 7894).

## Results

### Cells’ viability was maintained after 24 h shipment

The expanded hBMSC were transported within 24 hours from Oslo (Norway) to Bergen (Norway) at RT via DHL Express overnight courier. The majority of the cells cultured in different conditions expressed viability above 90%. Their viability on arrival at Bergen is summarised in Table 3.

**Table 3:** Viability of cells after shipment.

	Condition	Cell viability before shipment	Cell viability 24 h later
Donor I	Direct(AB + FGF_low_)	97%	85%
	ACK(AB + FGF_low_)	98%	91%
	Lympho(AB + FGF_low_)	98%	91%
	Direct(PL)	92%	84%
Donor II	Direct(AB + FGF_low_)	96%	94%
	Direct(AB + FGF_high_)	96%	93%
	Direct(PL)	97%	92%
Donor III	Direct(AB + FGF_low_)	98%	96%
	Direct(AB + FGF_high_)	98%	95%
	Direct(PL)	98%	92%

### hBMSC preserved variable degrees of stemness when cultured under different conditions

**Figure 1:**
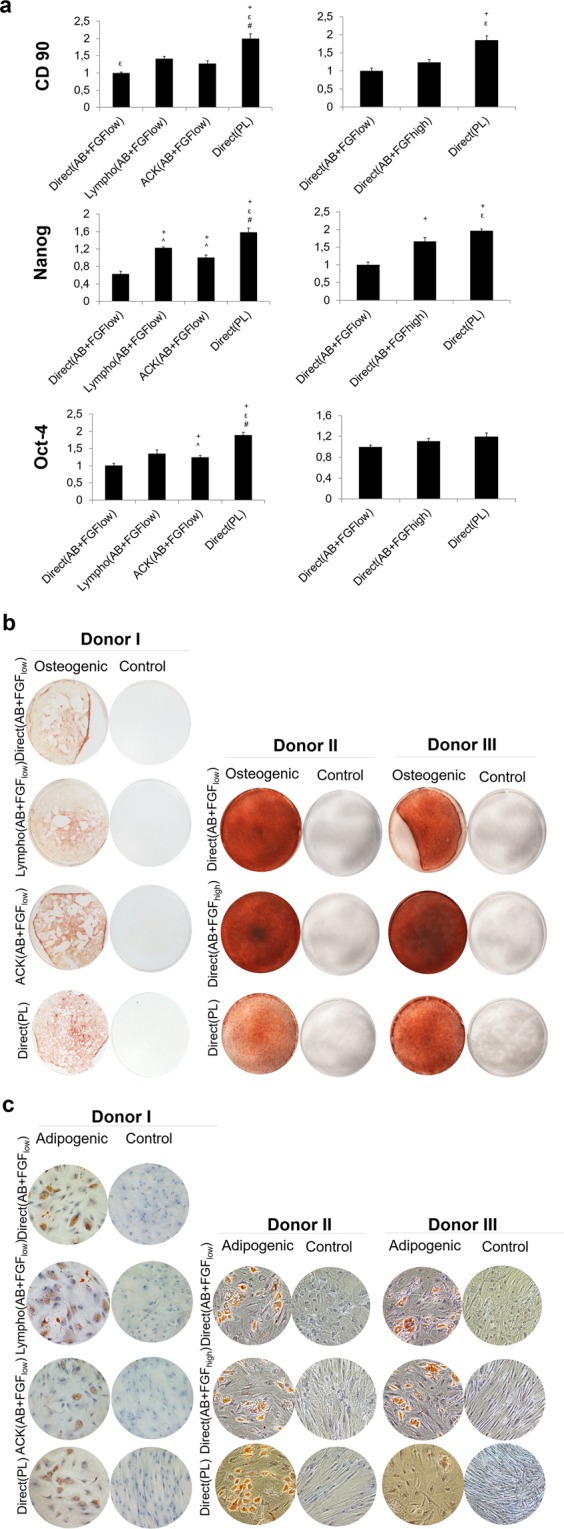
Multipotency characterisation *in vitro*. (**a)** Relative mRNA expression of MSC marker, CD90 and transcription factors regulating pluripotency, NANOG and Oct-4 after 1 week of non-induced culture after transport. Expression is presented relative to the Direct(AB + FGF_low_) group (p < 0.05). **(b)** Alizarin stain showing cells from all culture conditions and from all donors differentiated consistently into the osteogenic lineage when induced. **(c)** Oil red O stain confirming lipid vacuoles when adipogenic lineage was induced.

Undifferentiated hBMSC expressed significantly higher levels of CD90 mRNA when they were isolated directly from bone marrow and expanded in PL for all donors. The transcription factors regulating pluripotency, NANOG and Oct-4, were significantly upregulated in the cells that were isolated directly from bone marrow and expanded in PL for all donors, followed by Lympho(AB + FGF_low)_ in donor I and Direct(AB + FGF_high_) in donor II and III (Fig. 1a). Cells from all donors expanded under all different conditions tested showed multipotency when induced. The osteogenic differentiation was confirmed by presence of a mineralised matrix when stained for Alizarin red. Cells expanded with AB + FGF_high_ were observed to have the strongest Alizarin red stain, as assessed macroscopically (Fig. 1b). The adipogenic lineage was defined by the formation of lipid vacuoles stained by Oil red (Fig. 1c).

### Different isolation and culture conditions influenced the inflammatory responses *in vitro*

**Figure 2:**
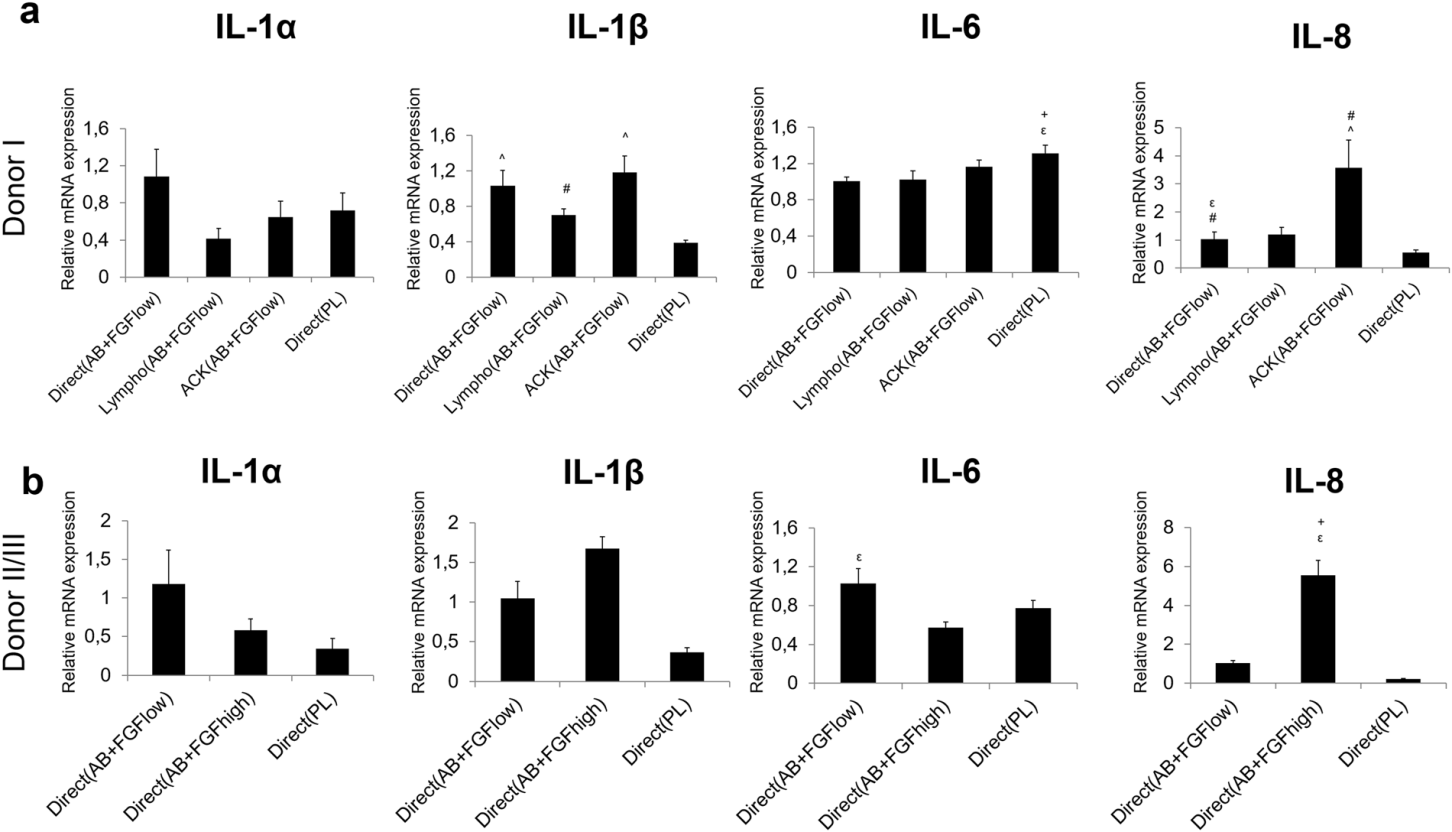
mRNA expressions of inflammatory markers *in vitro* 1 week post transport. Different groups evaluated from **(a)** donor I and **(b)** donor II/III. Y axes represent relative mRNA expressions for IL-1α, IL-1β, IL-6 and IL-8. Expression is presented relative to the Direct (AB + FGF_low_) group, (p < 0.05).

The mRNA of pro- and anti-inflammatory markers displayed variable expression among different groups. IL-1α, the pro-inflammatory marker showed the highest expression in Direct(AB + FGF_low_) for all donors **(**Fig. 2**)**. The lowest expression in donor I was detected in cells from Lympho(AB + FGF_low_) **(**Fig. 2a**)**. However, when only the Direct seeding method was compared for donor II and III, the lowest expression was seen in cells from Direct(PL) **(**Fig. 2b**)**. The lowest expression for IL-1β was presented by cells from Direct(PL) for all donors.

IL-6 showed the highest significant expression in cells from Direct(PL); however for donor I and III the cells from Direct(AB + FGF_low_) showed the highest expression, followed by cells from Direct(PL). The mRNA expression of IL-8 showed more consistency among the donors where cells expanded under AB + FGF portrayed the highest expressions.

### Different isolation and culture conditions influenced the osteogenic differentiation potential of the cells *in vitro*

**Figure 3:**
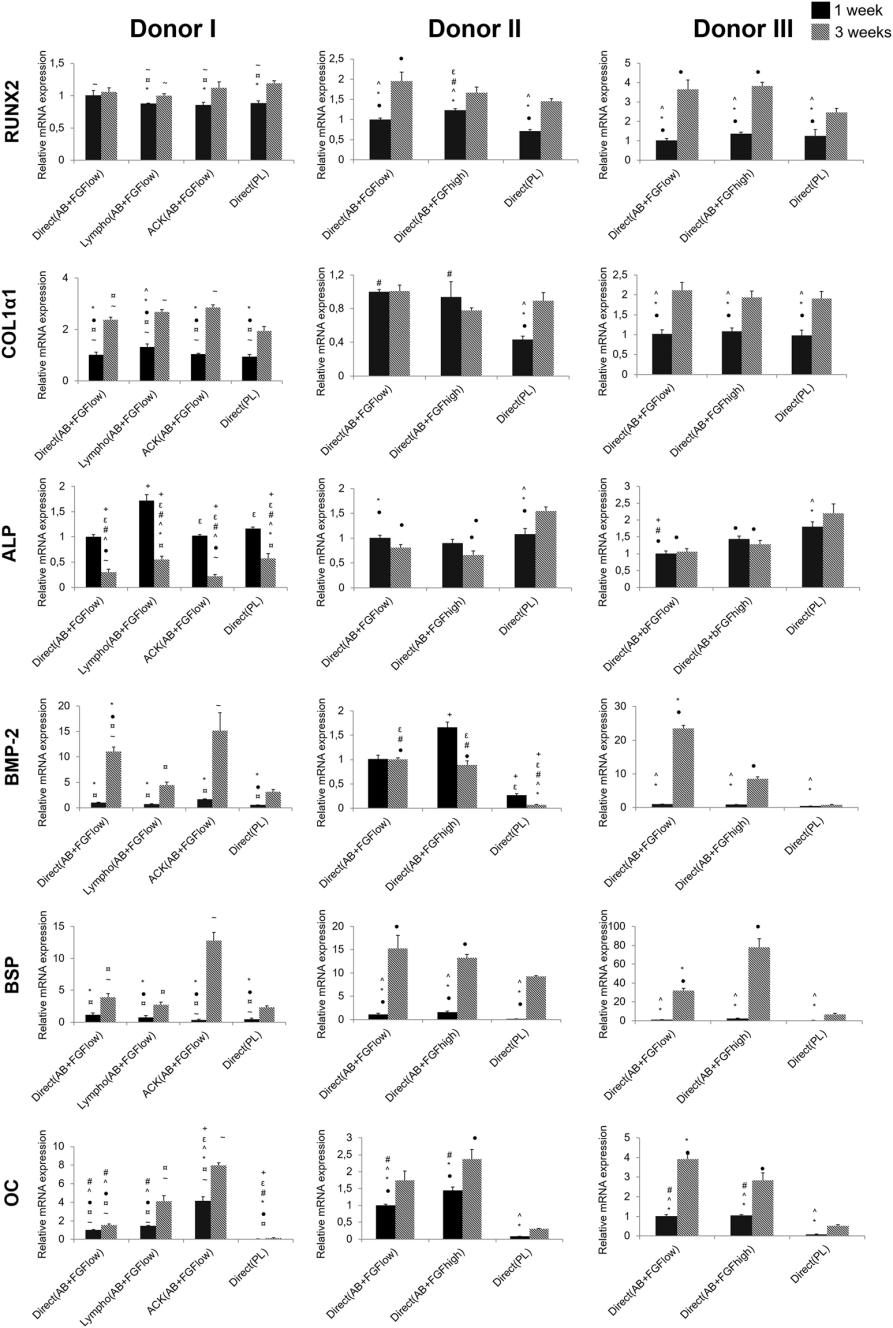
mRNA expressions of osteogenic markers *in vitro* after 1 and 3 weeks post transport. Y axes represent relative mRNA expressions for RUNX2, Col1α1, ALP, BMP-2, BSP and OC. Expression is presented relative to the Direct(AB + FGF_low_) group at 1 week, (p < 0.05).

The mRNA expression of most of the osteogenic markers showed generally comparable trends among all isolation and cell culture conditions tested here **(**Fig. 3**)**. The early transcription factor RUNX2 continued to be upregulated significantly from 1 to 3 weeks in all groups. After 1 week, cells isolated by direct method and cultured in AB + FGF showed the highest expression of RUNX2 for all donors. Early osteogenic marker, Col1α1, displayed a significant upregulation from 1 to 3 weeks in all groups except Direct(AB + FGF_high_) for donor II which showed an inversed trend. Generally, cells grown in AB serum, regardless of the isolation method showed higher expression at both time points compared to cells in PL.

ALP mRNA expression was significantly downregulated for all groups after 3 weeks except for Direct(PL), where it was upregulated in cells from donors II and III. The highest expression at both time points was in cells from Lympho(AB + FGF_low_) for donor I followed by cells from Direct(PL). For donor II and III, cells from Direct(PL) showed the highest significant expression at both time points.

The mRNA expression of BMP-2 showed a consistent significant upregulation after 3 weeks for donor I and donor III in all culture condition groups. However, a downregulation was observed after 3 weeks in donor II for most groups. The highest BMP-2 mRNA expression at both time points were seen from cells expanded in AB + FGF either by the ACK or Direct seeding method.

BSP mRNA presented comparable trends of expression among all groups for all donors. There was a significant upregulation after 3 weeks in all groups, with the highest expression from cells grown in AB + FGF in general and specifically ACK(AB + FGF_low_) for donor I. Osteocalcin, a late osteogenic marker, was significantly upregulated after 3 weeks along all culture condition groups for all donors. The highest osteocalcin mRNA expressions at both time points were seen from cells isolated and expanded in ACK(AB + FGF_low_) for donor I and similary cells grown in AB + FGF from donor II and III. Direct(PL) cells showed the lowest expression of BSP and osteocalcin at both time points for all donors.

### Different isolation and culture conditions influenced the adipogenic differentiation potential of the cells *in vitro*

**Figure 4:**
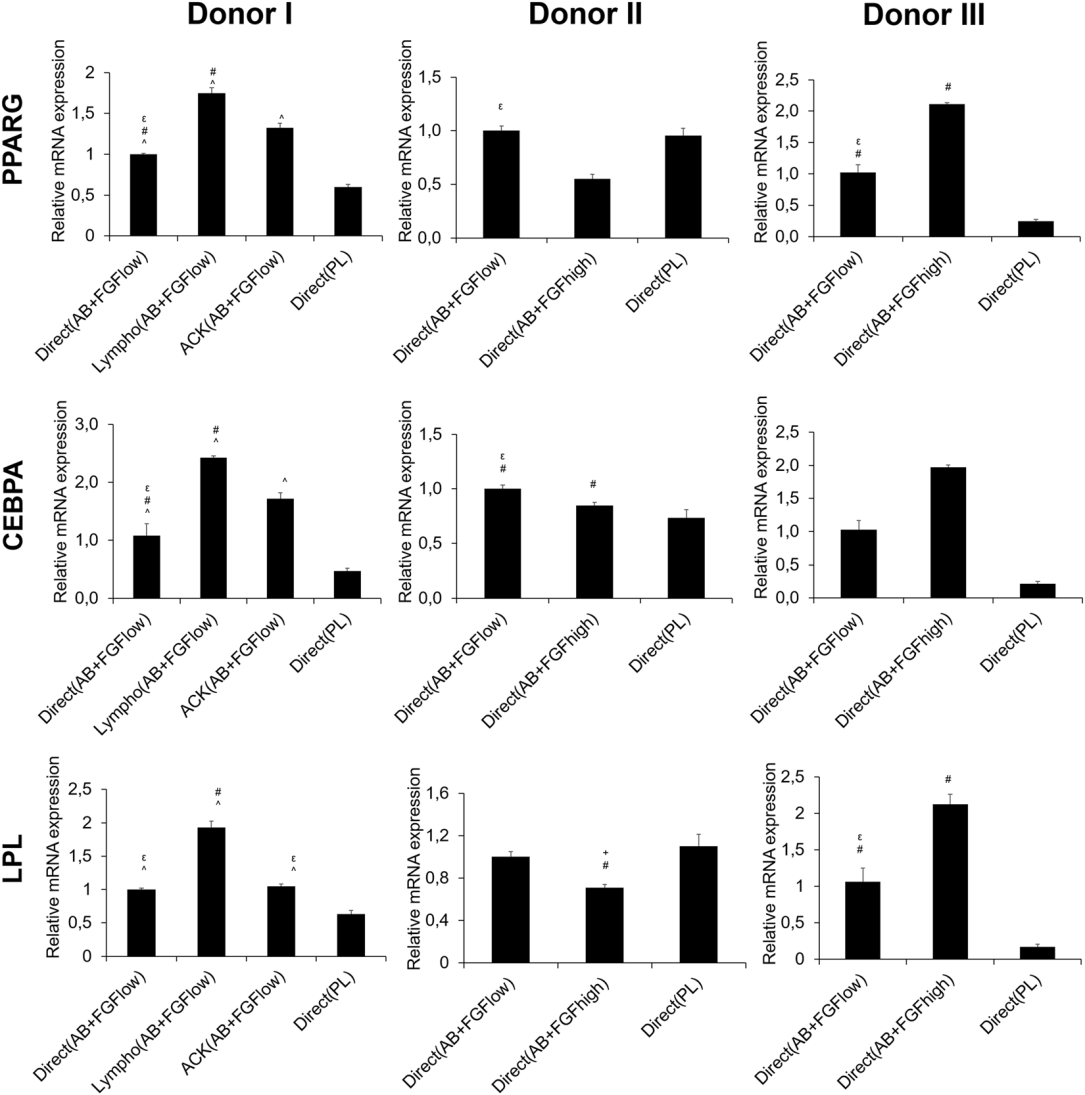
mRNA expressions of adipogenic markers *in vitro* after 2 weeks post transport. Y axes represent relative mRNA expressions for PPARG, CEBPA and LPL. Expression is presented relative to the Direct(AB + FGF_low_) group, (p < 0.05).

The mRNA expression of the adipogenic markers was evaluated at 2 weeks and it showed largely comparable trends among all isolation and cell culture conditions tested, proving the adipogenic differentiation and thus multipotency of the cultured hBMSC **(**Fig. 4**)**.

The highest expressions in all adipogenic markers (PPARG. CEBPA and LPL) were from Lympho(AB + FGF_low_) cells from donor I and the lowest expressions from Direct(PL) cells. Cells grown in AB + FGF in general from donor III expressed higher levels compared to Direct(PL) in all markers. Donor II showed variable expressions among the three markers between the groups.

### Different isolation and culture conditions influenced the replicative senescence of the cells

**Figure 5:**
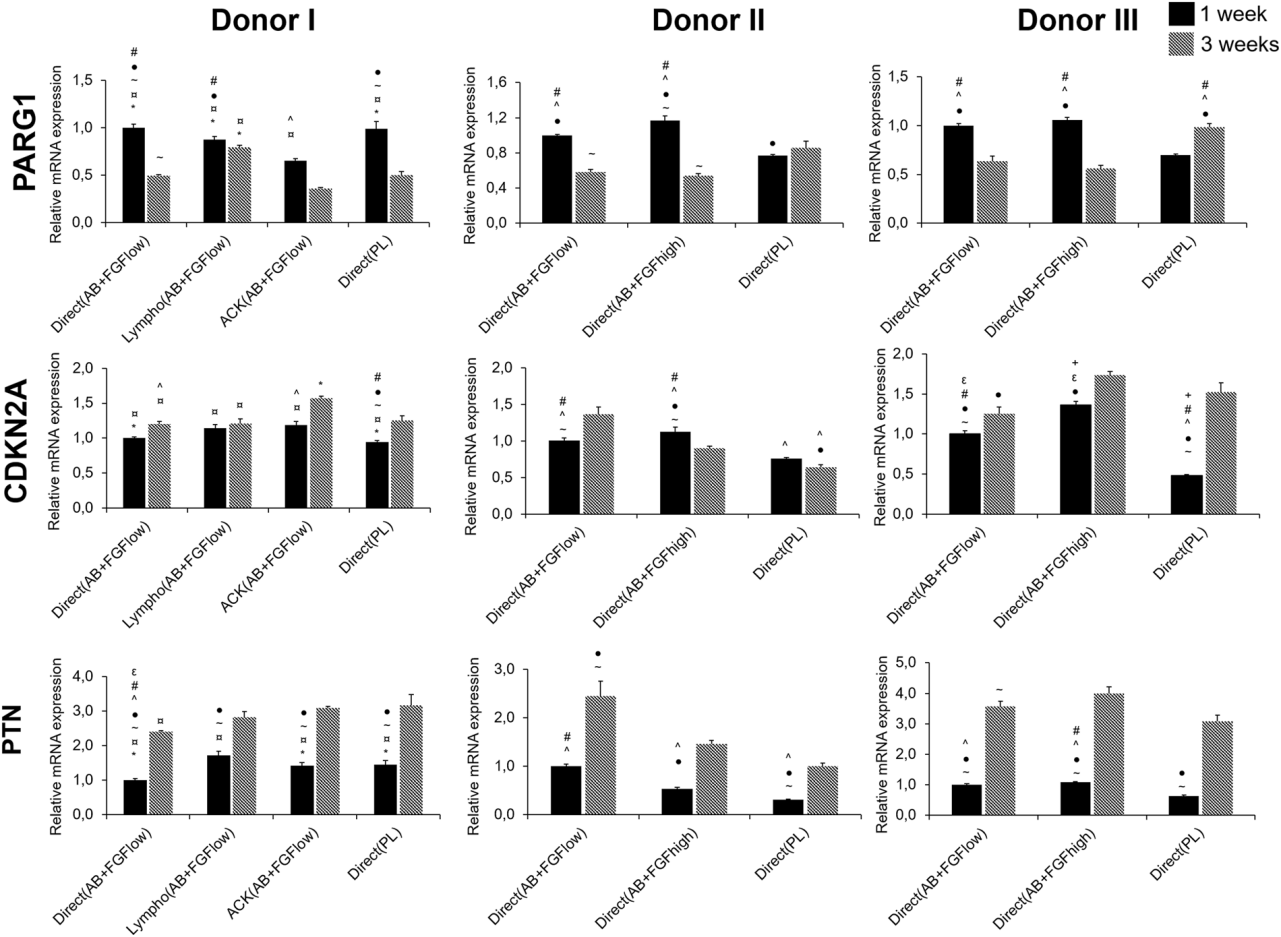
mRNA expressions of replicative senescence genes after 1 and 3 weeks in osteogenic culture. Y axes represent relative mRNA expressions for PARG1, CDKN2A and PTN. Expression is presented relative to the Direct(AB + FGF_low_) group, (p < 0.05).

The mRNA expression of a combination of the commonly upregulated and downregulated genes associated with cellular aging were evaluated after 1 and 3 weeks of osteogenically differentiated cells cultured in the different conditions. Significant differences were observed between the different expansion conditions and between time points **(**Fig. 5**)**. The mRNA expression of PTN at 1 week was comparable between all groups, however after 3 weeks there was an upregulation in all groups and significantly higher upregulation was observed in the AB + FGF groups in general in donor II and III. These results might elucidate the survival of Direct(AB + FGF_high_) *in vivo* up to 11 weeks compared to cells cultured in Direct(PL). PARG1 was significantly expressed lower in Direct(PL) in donor II and III in week 1 and CDKNA2 showed similarly the lowest expression significantly in Direct(PL) in all donors at week 1. After 3 weeks, Direct(PL) showed the highest expression of PARG1 in donor II and III and AB + FGF groups generally showed higher expressions in CDKNA2 **(**Fig. 5**)**.

### Different culture conditions influenced the inflammatory and host response potential of the cells *in vivo*

**Figure 6:**
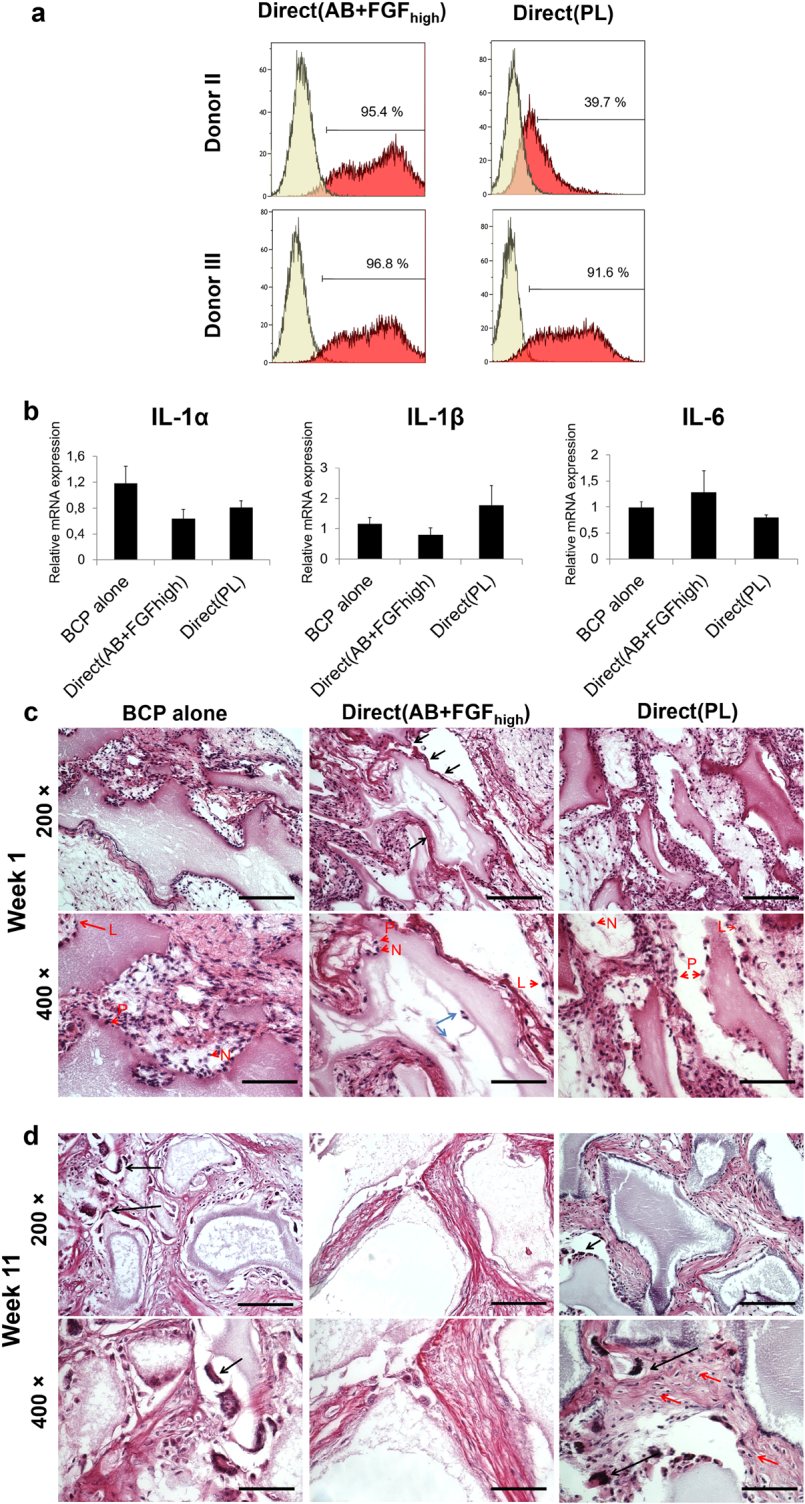
*In vivo* inflammatory and host responses. (**a**) Representative histograms of flow cytometry analysis showing HLA-DR expression on hBMSC pre-implantation; monoclonal antibody control (yellow) and the stained cells (red). (**b**) Mouse relative mRNA expressions 1 week post-implanting of the BCP and cell constructs *in vivo*. Y axes represent mRNA expressions for IL-1α, IL-1β, IL-6 (p < 0.05). (**c**) Histological micrographs after 1 week of the different groups implanted. Magnification 400× showing the different inflammatory cells recruited. L - lymphocytes, N - neutrophils, P - plasma cells. (**d**) Histological micrographs after 11 weeks showing recruited foreign body giant cells (black arrows) and a cellular connective tissue with scattered fibroblast-like cells in Direct(PL) (red arrows). Scale 100 µm.

Before *in vivo* implantation, cells from Direct(AB + FGF_high_) and Direct(PL) showed comparable expressions of all the tested surface cell markers (data not shown) except for HLA-DR + where the percentage and intensity reduced considerably in cells from Direct(PL) for both donors II and III (Fig. 6a**)**. Implanted hBMSC did not express any human inflammatory genes *in vivo* (data not shown). Mouse inflammatory responses showed an upregulation of IL-1α and IL-1β expression in Direct(PL) **(**Fig. 6b). The marker IL-6 was upregulated in cells from Direct(AB + FGF_high_) and there was no expression for IL-8.

Histologically, inflammatory cells (neutrophils, plasma cells and lymphocytes) infiltrated all implants in the first week (Fig. 6c red arrows). More inflammatory cells were detected in Direct(AB + FGF_high_) and Direct(PL). Fibroblast-like cells were visible within a dense connective tissue close to the granules in Direct(AB + FGF_high_) and Direct(PL), compared to the interlaced areas of loose connective tissue observed in BCP alone. Thick and organised layers of collagen bordering the granules with cells from Direct(AB + FGF_high_) group **(**Fig. 6c black arrows)were observed as compared to cells from BCP alone and Direct(PL) group. Peripheral resorption/degradation of the granules was greater in Direct(AB + FGF_high_) compared to the control and to Direct(PL) and cells could be identified within the pores of the granules in Direct(AB + FGF_high_) (Fig. 6c blue arrows).

After 11 weeks *in vivo* multi-nucleated giant cells were seen in close contact to the peripheries of the granules in all implanted groups mostly in the BCP alone and Direct(PL) groups **(**Fig. 6d black arrows). Integration of the granules with the surrounding tissues appeared to be less obvious, with a decrease in granules’ surface area, indicative of accelerated resorption than at 1 week. The granules in Direct(AB + FGF_high_) continued to degrade faster, thus the less visible multi-nucleated giant cells. Granules were situated between areas of dense and disperse connective tissue, with the connective tissue closer to the granules being more dense in the Direct(AB + FGF_high_) and Direct(PL) groups compared to the control. Cellular connective tissue with scattered fibroblast-like cells was more prominent in Direct(PL) (Fig. 6d red arrows).

### *In vivo* osteogenic potential showed variability with different culture conditions

After 1 week there were no expressions for mice osteogenic genes; however the human genes from hBMSC were expressed after implantation. All osteogenic markers evaluated, RUNX2, COL1α2, ALP showed a consistent significant upregulation in the Direct(AB + FGF_high_) group compared to the control and Direct(PL).

**Figure 7:**
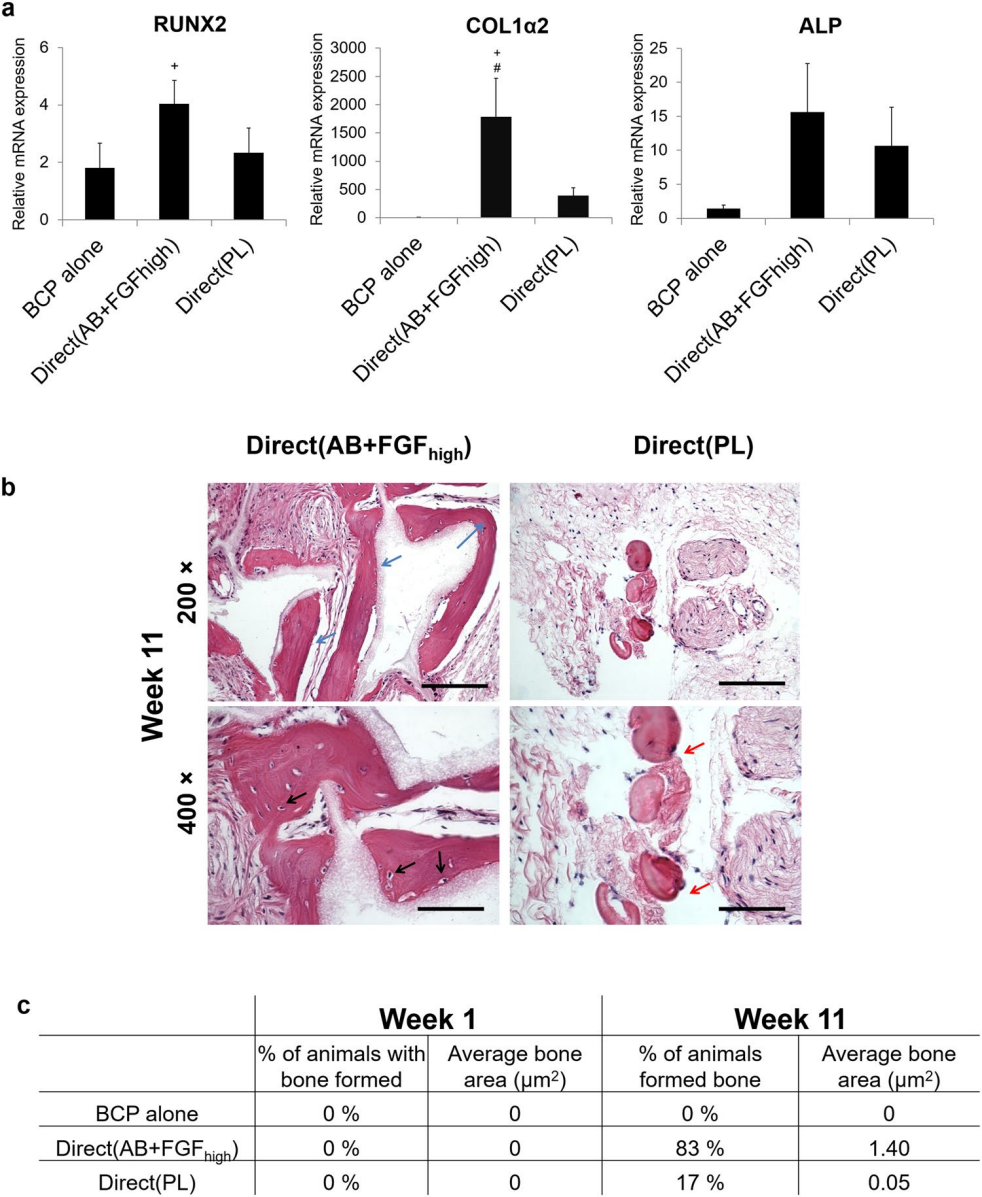
mRNA expression of human osteogenic markers *in vivo* and *de novo* bone formation. (**a)** Relative mRNA expressions after 1 week *in vivo*. Y axes represent relative mRNA expressions for RUNX2, COL1α2 and ALP relative to endogenous reference GAPDH. Expression is presented relative to BCP alone (p < 0.05). **(b)** Histological micrographs after 11 weeks showing mature mineralised ectopic bone (blue arrows), osteocyte lacunae in Direct(AB + FGF_high_) explants (black arrows) and immature bone-like regions in Direct(PL) (red arrows). Scale 100 µm. **(c)** Histomorphometrical analyses of the frequency and quantity of bone formed in the different implant groups after 1 and 11 weeks.

Histological sections examined for mineralisation showed that after 11 weeks *in vivo*, the frequency, maturation and quantity of ectopically formed bone were different among the different groups. Cells from Direct(AB + FGF_high_) formed mature mineralised ectopic bone in 83% of the animals, compared to immature mineralised tissues formed in only 17% of the animals implanted with Direct(PL). BCP alone showed no signs of mineralisation, however there were areas of dense collagen seen around the granules. Osteocytes in lacunae were found within the bone like structures in Direct(AB + FGF_high_) (Fig. 7b black arrows). Also, cuboidal osteoblast-like cells were seen surrounding the peripheries of the granules and bordering the newly formed bone.

The Direct(PL) group histologically contained more areas of dense collagen and the de novo-formed bone appeared to have a higher ratio of mineralised immature ‘bone-like’ regions (Fig. 7b red arrows).

### Identifying implanted human BMSC *in vivo*

**Figure 8:**
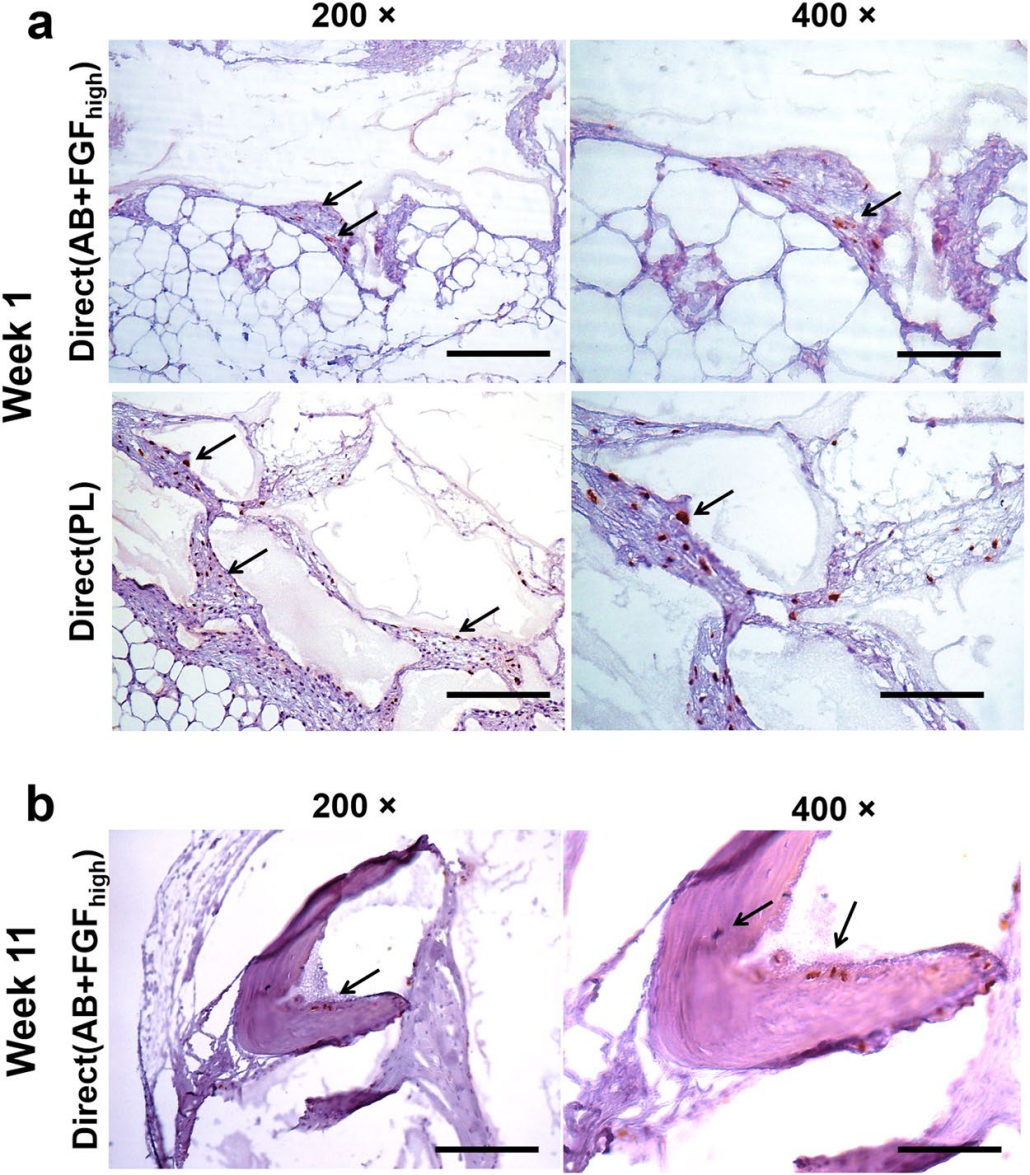
Human cell identification by *in situ* hybridisation of the human-specific repetitive Alu sequence. (**a)** Micrographs showing human cells 1 week post-implantation populated with host cells (purple nuclei) and human cells (brown nuclei) surrounding BCP granules in both Direct(AB + FGF_high_) and Direct(PL) (black arrows). Human cells populating dense connective tissue in Direct(PL) compared to a loose connective tissue in Direct(AB + FGF_high_). **(b)** Human cells identified by the brown nuclei (black arrows) in osteocyte lacunae (black arrows) within the mineralised bone formed in Direct(AB + FGF_high_) after 11 weeks post implantation. Scale 100 µm.

*In situ* hybridisation using the human-specific repetitive Alu sequence demonstrated that i*n vivo* explants were populated primarily by host connective tissue cells (fibroblasts) depicted by the purple nuclei and purple fibers. Human cells were identified with brown nuclei and were observed after week 1 in both Direct(AB + FGF_high_) and Direct(PL) groups **(**Fig. 8a black arrows). At week 1, human cells in Direct(PL) were seen to infiltrate a dense connective tissue that is organised and wrapped around the periphery of intact BCP granules. On the contrary, the human cells in Direct(AB + FGF_high_) were observed to populate a looser connective tissue (less organised) that is surrounding BCP granules that are less integrated. A similar picture of loose connective tissue with less human cell population was observed surrounding the less integrated BCP granules in Direct(PL) explants. Morphologically, no differences were observed between the human cells infiltrating in both groups. After 11 weeks human cells were detected in the explants from Direct(AB + FGF_high_) only **(**Fig. 8b black arrows). The human cells were found embedded in osteocyte lacunae within the mineralised bone formed ectopically with no cells found in the surrounding connective tissue. No inflammatory cells were identified infiltrating the connective tissue or bone which is populated with human cells.

## Discussion

In this study, we compared different isolation conditions of MSC from human bone marrow and expansion in different ‘humanised’ media, specifically, human AB serum (AB) supplemented with FGF2 or PL. Several studies have confirmed that human derivatives can substitute for FBS in expansion of MSC^26,27^ and therefore this comparison was not the objective of the current study. Clinical applications of MSC require a cell number that cannot be provided by simple bone marrow aspiration, therefore, an *ex vivo* expansion step is inevitable. However, their clinical applications may be limited by the ability to expand their cell numbers *in vitro* while maintaining their differential potentials and stem cell properties/‘stemness’.

In a GMP system, cells are produced under the highest quality requiring product reproducibility^6,7^. Cell survival and quality assurance are essential aspects to be considered in addition to the therapeutic potency. Taking this into consideration, the viability of our cells after shipment was considered comparable to that before shipment, hence successful. However, in some occasions, the GMP facilities are not in close proximity to the clinical centre where the cell therapy is taking place. The ability to preserve MSC phenotype and function in a sterile condition until the confirmation of an absence of bacterial contamination is important for quality control prior to clinical transplantation^28^. Dimethylsulfoxide (DMSO) and FBS are commonly used as cryoprotectants. However, the use of DMSO can result in toxicity while animal proteins present in serum-supplemented culture media, can induce immune responses after transplantation. Cryopreserving the MSC in xeno-free can promote cell therapy ‘off the shelf’, but the cryostorage protocols need to evaluated thoroughly to test the resiliency of the cells. This has been effectively presented with autologous plasma used as a cryoprotectant in combination with DMSO, where it preserved human MSC’s therapeutic efficacy after rapid thawing and implantation^28^. However, it has been reported that the cells’ behaviour can be affected by different cryoprotectants^29^. Platelet lysate as a cryoprotectant revealed comparable efficacy compared to FBS^30^, however how the different types of human-derived cryoprotectants preserve MSC need to be compared in a bone regeneration context. In our results (Table 3), cells expanded in Direct(PL) showed the highest viability in Donor II and III, although this higher viability, compared to the other groups, was not conserved after 24 h of shipment in room temperature. Moreover, we have previously shown that expansion medium supplemented with PL allowed bone marrow MSC to continue proliferating steadily for more than 120 days^31^. Therefore, due to the presence of high amounts of growth factors and cytokines, particularly PDGF in PL^16^, which has an anti-apoptotic effect on progenitor cells^32^, we could conjecture that PL is a cryoprotectant that could preserve the high viability and proliferative capacity expressed during thawing and expansion. However, success of survival after engraftment is not necessarily a similar reflection.

Concerns that the monolayer expansion phase of MSC results in loss of multipotency, the ability to self-renew, and promotes a tendency toward osteogenesis have been previously reported^33^. In our study, hMBCs before differentiation were analysed for presence of pluripotency markers Oct-4 and NANOG, which are reported to function in coherence as key transcription factors for the pluripotent and self-renewing phenotypes of undifferentiated embryonic stem cells^34,35^. However, their role in the self-renewal and undifferentiation of MSC is controversial with postulated mechanisms behind their role in maintaining MSC in an undifferentiated state^36^. Interestingly, the MSC expanded in PL were highly positive for Oct-4 and NANOG mRNA, therefore, PL maintained primitive MSC during expansion, compared with that in AB serum. Similarly, a study using PL showed that MSC preserve their multipotency with elevated expressions of Oct-4 and NANOG compared to when FBS is used to expand MSC^37^. This can be considered advantageous since this will likely affect the functional outcomes following *in vivo* transplantation. Compared with other human supplements, PL was reported to be the human-derived supplement of choice for expanding hBMSC in recent clinical trials^22^. Additionally, CD90, a marker expressed by bone marrow subpopulations of CFU-F^38^, was upregulated in Direct(PL) for all donors and least expressed by Direct(AB + FGF_low_) for all donors, which highlights the role played by PL in preserving the MSC multipotency. Nevertheless, many studies have shown that expansion of MSC in different types of media can lead to increased heterogeneity and enrichment of certain subpopulations, which can affect cellular genotypes and phenotypes^39^. Reports have shown that addition of PL to the medium produces MSC with a reduced adipogenic differentiation potential, whereas other studies have shown that it favours osteogenesis and chondrogenesis^40–42^. These conflicting outcomes may be attributed to inconsistency in preparation procedures as well as the effects of PL donors of different ages^43^.

Our results also showed variability in the expression of the stemness markers among the groups where hBMSC were expanded in AB + FGF but using different isolation methods. This highlights the additional role played by isolation methods in the cell phenotype/genotype. MSC are isolated from bone marrow using different methods by different laboratories; these include directly plating the whole bone marrow, density centrifugation, red blood cell lysis and magnetic/fluorescence activated cell sorting. In our study, one of the methods used to isolate MSC was processing by density centrifugation, using Lymphoprep density media. In donor I, Lympho(AB + FGF_low)_ portrayed the second highest expression for stemness markers after Direct(PL). Density centrifugation separates the mononuclear cell fraction from the other cellular or non-cellular constituents in bone marrow aspirate (*e.g*. red blood cells). The resulting mononuclear cells include a mixed population, such as T cells, B cells monocytes, hematopoietic stromal cells and MSC. The density centrifugation process itself reduces the total yield of mononuclear cells from bone marrow^44^, but may increase the colony forming unit fibroblasts (CFU-F) efficiency compared to direct plating/seeding and ACK lysis methods^45^. This can explain their higher expressed stemness. Inter-donor variations cannot be avoided, however, to rule out variations that have been demonstrated when comparing the phenotype of MSC population derived from two serial bone marrow aspirates from the same person^46^, the same bone marrow sample was divided and processed differently in this study.

In our AB serum, it was supplemented with FGF2, and purified growth factors, such as FGF2, are added to culture conditions even in the presence of FBS or blood-derived supplements to enhance proliferation and overall behaviour^47^. FGF2 is a known mitogen of MSC, also functioning to maintain multipotency and to promote subsequent differentiation *in vitro*^47,48^. However, their effects can greatly vary depending on their concentrations and interactions, as portrayed by our results. The concentration of FGF-2 was doubled, but that increase was not reflected directly on the expression of osteogenic genes for example. Concentrations, not standard, up to 10 ng/ml of FGF2 are reported in the literature, causing difficulties when attempting to compare studies.

*In vitro* inflammatory gene expressions were evaluated in undifferentiated hBMSC to evaluate the role played by the isolation and expansion conditions solely. The pro-inflammatory marker IL-1α was least expressed in Lympho(AB + FGF_low_). This method compared to others involves the selection of the bone marrow mononuclear cells and it is in line with a previous report where directly seeding complete bone marrow or using methods not involving selecting mononuclear cells preserved the immunomodulatory capacity of MSC^49^. IL-6 and IL-8 are inflammatory cytokines that play important roles in osteogenic differentiation for bone regeneration and remodelling^50,51^. Our results showed the highest expression of IL-6 from Direct(PL) in donor I and the second highest in donor II and III. The secretion of IL-6 was shown to be increased by MSCs cultured with PL compared with FBS^52^ and IL-6 has been reported to maintain MSC stemness^53^. Considering that Direct(PL) cells showed a relatively late osteogenic differentiation, this explains the reverse expressions of IL-8, since in bone regeneration this marker is involved with osteoclast function which is in late stages of remodelling^50^. MSC have the ability to express HLA-II when exposed to inflammatory stimulants^54^ and these stimulations trigger their immunosuppressive function^55^. HLA-II antigens are recognised by CD4 + T lymphocytes, and MSC avoid immune rejection by immunomodulation of the local environment^56^. HLA-DR is an MHC class II cell surface receptor, and a stronger expression of HLA-DR compromises the immune privilege of MSC^57^. Direct(PL) cells showed a lower expression of HLA-DR compared to Direct(AB + FGF_high_), which correlates with the more ‘stemness’ phenotype expressed from Direct(PL) cells and their higher expression of anti-inflammatory marker IL-6 *in vitro* and reduced pro-inflammatory markers (IL1α and β). It has been reported that the HLA-II expression in MSC displays an inversely proportionate relation with the stemness of the cells^58^.

Nevertheless, the impact of HLA-DR expression on MSC potency and function remains controversial due to its dynamic expression from MSC in culture with time^56^. Different culture conditions (human serum versus platelet lysate) were not found to affect the HLA-DR expression from BMSC^56^, however addition of FGF was reported to express higher HLA-DR in BMSC compared to when cells were cultured in platelet lysate^59^, in line with our results. The expression of HLA-DR has been suggestive of differentiation commitment^56^, which can explain the osteogenic potential of cells from AB + FGF *in vitro* and *in vivo*.

*In vivo*, when the hBMSC were implanted, no human inflammatory markers were detected at mRNA level, however the implanted cells posed varying inflammatory reactions from the host cells between groups.

Our *in vitro* osteogenic differentiation results showed mostly elevated expressions of osteogenic markers when cells were generally expanded in AB + FGF2. It has been reported previously that MSC cultured in FBS in combination with FGF-2 showed a superior growth compared to PL^49^. However, a closer look into when the different isolation methods were compared in donor I, a significant difference was portrayed even when the cells were expanded in the same media (AB + FGF2). The bone markers BMP-2, BSP and osteocalcin showed significantly higher expressions after 3 weeks in the ACK(AB + FGF_low_). This can be attributed as well to the fact that other isolation methods, such as density centrifugation increase the sub-population of naive MSC more prone to self-renewal than differentiation^45^.

MSC expanded in PL showed relatively higher expressions in the early bone markers, such as RUNX2 and ALP. This compliments the high stemness markers that were expressed which propose the late differentiation of the cells in this condition. A previous study reported that addition of FGF-2 in the culture medium resulted in reduction in expression of ALP^60^, which could be a postulation for our higher ALP expressions from Direct(PL) in donor II and III. Besides the naive MSC state in the Direct(PL), another factor for the lesser osteogenic potential could be the addition of heparin in the medium to prevent gel formation. It was shown that a relatively high concentration of heparin in culture media supplemented with human PL compromises proliferation as well as adipogenic and osteogenic differentiation of MSC^61^.

Characterising hBMSC performance and potency as a therapeutic entity requires additional tests for *in vivo* differentiation potential. Based on the *in vitro* results, the direct seeding method was chosen to go further. This helps to reduce confounding factors and establish methods easy to standardise with least manipulation for the bone marrow aspirates. As previously reported, MSC expanded in FBS are able to undergo bone formation when implanted on BCP scaffolds^62^, however it is important to define that this characteristic and quintessential multipotent MSC phenotype are not lost with the optimised humanised culture conditions proposed in this study.

*In vivo*, hBMSC expressed the osteogenic genes (RUNX2, COL1α2 and ALP) when expanded in Direct(AB + FGF_high_) and showed a higher frequency of bone formation too, which reflects cell survival and differentiation, indicating a direct contribution of the implanted hBMSC in bone formation. Interestingly, the *de novo* bone in Direct(PL) seemed to have immature regions with more disorganised collagen, suggestive of woven bone morphology and indicating tissue-formation processes were still continuing after 11 weeks of implantation. This can be reflected on the higher self-renewing potential of the cells in this group that was depicted *in vitro*. When observed closely, the collagen of the new bone in the Direct(AB + FGF_high_) group appeared much more organised with residing osteocytes within lacunae, suggestive of lamellar structure. Several studies suggest a direct contribution of MSC to regenerate bone as they differentiate into osteoblastic lineage when implanted locally, and contrary studies postulate that the implanted MSC exert a paracrine effect on host cells thus home circulating hematopoietic progenitors^63^ and endogenous osteogenic progenitor cells^64^. The *in situ* hybridisation results suggest the cells from the different groups might have contributed differently when forming bone. In Direct(AB + FGF_high_) human cells were still identified after 11 weeks, located in the lacunae of mature mineralised bone, which was not the case for Direct(PL). It could be that the less differentiated cells from Direct(PL) released cytokines and growth factors to recruit endogenous progenitor cells. Although no human cytokines were detected from the explants, however the mouse pro-inflammatory markers detected were highly expressed in this group. Therefore, the host inflammatory environment induces the recruitment and the homing of MSC^65^. IGF-1 and PDGF that are reported to play a role in homing MSC^66^ are major constituents of PL^16^.

Replicative senescence is reflected by significant morphological changes; cellular enlargement, debris and vacuoles intracellulary leading to a cease of proliferation^67^. The cells in our study were expanded for only 2 passages before they were used in the *in vitro* and *in vivo* experiments. No senescent/related morphological changes or phenotypical changes were identified when the cells were routinely observed macroscopically. However, previous reports have shown that replicative senescence of MSC is a continuous process starting from the first passage^67^, and this process includes, in addition to alterations in phenotype and differentiation potential, senescence-associated gene expression changes. Therefore, to reflect the heterogeneity in the cellular aging process, global gene expression patterns have been evaluated and still constantly identified. PTN demonstrated to play important roles in survival and self-renewal of human embryonic stem cells and retention of bone marrow hematopoietic stem cells. Its down-regulation was shown to be associated with decline of proliferation capacity of senescent MSC^68^. The mRNA expression of PTN after 3 weeks with a significantly higher upregulation in the AB + FGF groups in general in donor II and III could elucidate the survival of Direct(AB + FGF_high_) *in vivo* up to 11 weeks compared to cells cultured in Direct(PL). The trend was reflected in the other markers, PARG1 and CDKNA2, cell cycle inhibitors that were downregulated in Direct(PL) in most donors in week 1. Replicative senescence-associated gene expression changes in hBMSCs isolated in different methods and expanded under xenogenic and xeno-free culture conditions demonstrated high similarity^68^. From our results, an effect of culture conditions on the senescence of the cultured hBMSC could be detected and an explanation for the *in vivo* cell survival and *in vivo* performance of the different groups can be postulated. However, several factors can be considered that influence the variation on gene expressions *in vitro*, such as donor age and cell density.

In addition to the isolation methods, the differing behaviours of MSC can be ascribed to the different cytokine contents of AB serum and PL. Also this might mean that 10% supplement of each is not a standard comparison as it may seem. It has been documented, for example, that age-related differences in human serum and PL composition occur and have a direct effect on MSC performance^43^; however, our results are related to commercially available allogeneic serum pooled from many hundred donors and PL pooled from 76 donors. A combination of human serum and PL to supplement culture medium showed an improved proliferation of MSC compared to when expanded in only human serum^21^.

In our results, Direct(AB + FGF_high_) cells expressed superior osteogenic potential *in vivo*, and these cells were identified until 11 weeks *in vivo*. This indicates that the poor survival of transplanted cells is a limitation for clinical regeneration that depend on long-term engraftment. A better understanding of how the cytokines present in inflammatory environments *in vivo* moderate MSC could be useful to develop more effective priming strategies to enhance MSC survival and subsequent therapeutic efficacy.

Coupling GMP xeno-free MSC production with the manufacturing of primed MSC can be considered as complementary strategies to enhance cell survival and therapeutic efficacy. Potential mechanisms of MSC primed therapeutics include gene editing^69^ to engineer MSC and thus promoting tissue regeneration through cell differentiation^70^. Moreover, via the delivery of bioactive factors through different secretory modes including *ex vivo* cell engineering^71^ or the production of induced MSC from induced pluripotent stem cells and preserve immunomodulatory characteristics in addition to enhanced cell survival and proliferation^57^.

## Conclusions

To fully recognise and exploit the therapeutic potential of MSC, an inclusive evaluation of their stemness, lineage, cell surface markers and transcription factors, in line with their isolation and expansion was required. In our work, we evaluated the effects of isolation methods and ‘humanised’ culture conditions on the potency of bone marrow derived MSC. Taken together, our results showed a significant effect of the isolation method and demonstrated a relatively consistent pattern of efficacy from 3 donors, and portrayed a tendency of hBMSC expanded in PL to retain a more stem-like phenotype which elucidates their delayed differentiation and different inflammatory expressions.

## Supplementary information

Supplementary Dataset 1

## Data Availability

Data will be made available on request.

## References

[1] Gomez-Barrena E (2011). Bone regeneration: stem cell therapies and clinical studies in orthopaedics and traumatology. J Cell Mol Med.

[2] Ullah I, Subbarao RB, Rho GJ (2015). Human mesenchymal stem cells-current trends and future prospective. Bioscience reports.

[3] Brennan, M. A. *et al*. Inferior *In Vivo* Osteogenesis and Superior Angiogeneis of Human Adipose-Derived Stem Cells Compared with Bone Marrow-Derived Stem Cells Cultured in Xeno-Free Conditions. **6**, 2160–2172 (2017). 10.1002/sctm.12274 PMC5827746 29485758

[4] Kim N, Cho SG (2013). Clinical applications of mesenchymal stem cells. Korean J Intern Med.

[5] Li, T. & Wu, Y. Paracrine molecules of mesenchymal stem cells for hematopoietic stem cell niche. *Bone marrow research* **2011** (2011). 10.1155/2011/353878 PMC3196250 22046560

[6] Rohde, E., Schallmoser, K., Bartmann, C., Reinisch, A. & Strunk, D. GMP‐Compliant Propagation of Human Multipotent Mesenchymal Stromal Cells. Pharmaceutical Sciences Encyclopedia: Drug Discovery, *Development, and Manufacturing*, 1–20 (2010).

[7] Hart ML, Brun J, Lutz K, Rolauffs B, Aicher WK (2014). Do we need standardized, GMP-compliant cell culture procedures for pre-clinical *in vitro* studies involving mesenchymal stem/stromal cells?. Journal of Tissue Science & Engineering.

[8] Antunes M, Pottering H (2007). Regulation (EC) No 1394/2007 of The European Parliament and of The Council of 13 November 2007 on advanced therapy medicinal products and amending Directive 2001/83/EC and Regulation (EC) No 726/2004. J. Eur. Union.

[9] Tekkatte, C., Gunasingh, G. P., Cherian, K. & Sankaranarayanan, K. “Humanized” stem cell culture techniques: the animal serum controversy. *Stem cells international* **2011** (2011). 10.4061/2011/504723 PMC3096451 21603148

[10] Jin X, Xu Q, Champion K, Kruth HS (2015). Endotoxin contamination of apolipoprotein AI: effect on macrophage proliferation–A cautionary tale. Atherosclerosis.

[11] Cimino, M., Gonçalves, R., Barrias, C. & Martins, M. Xeno-free strategies for safe human mesenchymal stem/stromal cell expansion: supplements and coatings. *Stem cells international* **2017** (2017). 10.1155/2017/6597815 PMC5660800 29158740

[12] Shahdadfar A, Frønsdal K, Haug T, Reinholt FP, Brinchmann JE (2005). *In vitro* expansion of human mesenchymal stem cells: choice of serum is a determinant of cell proliferation, differentiation, gene expression, and transcriptome stability. Stem cells.

[13] Abdallah BM, Haack-Sørensen M, Fink T, Kassem M (2006). Inhibition of osteoblast differentiation but not adipocyte differentiation of mesenchymal stem cells by sera obtained from aged females. Bone.

[14] Witzeneder K (2013). Human-derived alternatives to fetal bovine serum in cell culture. Transfusion Medicine and Hemotherapy.

[15] Mannello F, Tonti GA (2007). Concise review: no breakthroughs for human mesenchymal and embryonic stem cell culture: conditioned medium, feeder layer, or feeder‐free; medium with fetal calf serum, human serum, or enriched plasma; serum‐free, serum replacement nonconditioned medium, or ad hoc formula? All that glitters is not gold!. Stem cells.

[16] Burnouf T, Strunk D, Koh MB, Schallmoser K (2016). Human platelet lysate: replacing fetal bovine serum as a gold standard for human cell propagation?. Biomaterials.

[17] Doucet C (2005). Platelet lysates promote mesenchymal stem cell expansion: A safety substitute for animal serum in cell‐based therapy applications. Journal of cellular physiology.

[18] Rauch C (2011). Alternatives to the use of fetal bovine serum: human platelet lysates as a serum substitute in cell culture media. ALTEX-Alternatives to animal experimentation.

[19] Jonsdottir-Buch SM, Lieder R, Sigurjonsson OE (2013). Platelet lysates produced from expired platelet concentrates support growth and osteogenic differentiation of mesenchymal stem cells. PLoS One.

[20] Schallmoser, K. & Strunk, D. In Basic Cell Culture Protocols 349–362 (Springer, 2013).

[21] Brun J, Abruzzese T, Rolauffs B, Aicher WK, Hart ML (2016). Choice of xenogenic-free expansion media significantly influences the myogenic differentiation potential of human bone marrow–derived mesenchymal stromal cells. Cytotherapy.

[22] Gjerde C (2018). Cell therapy induced regeneration of severely atrophied mandibular bone in a clinical trial. Stem Cell Res Ther.

[23] Schallmoser, K. & Strunk, D. J. J. O. V. E. J. Preparation of pooled human platelet lysate (pHPL) as an efficient supplement for animal serum-free human stem cell cultures (2009). 10.3791/1523 PMC3164065 19881465

[24] Mohamed-Ahmed S (2018). Adipose-derived and bone marrow mesenchymal stem cells: a donor-matched comparison. Stem Cell Res Ther.

[25] Brennan MÁ (2014). Pre-clinical studies of bone regeneration with human bone marrow stromal cells and biphasic calcium phosphate. Stem cell research & therapy.

[26] Mojica-Henshaw, M. P. *et al*. Serum-converted platelet lysate can substitute for fetal bovine serum in human mesenchymal stromal cell cultures. **15**, 1458–1468 (2013). 10.1016/j.jcyt.2013.06.014 24199591

[27] Shanbhag, S., Stavropoulos, A., Suliman, S., Hervig, T. & Mustafa, K. J. T. E. P. B. R. Efficacy of humanized mesenchymal stem cell cultures for bone tissue engineering: a systematic review with a focus on platelet derivatives. **23**, 552–569 (2017). 10.1089/ten.TEB.2017.0093 28610481

[28] Chin S-P (2010). Cryopreserved mesenchymal stromal cell treatment is safe and feasible for severe dilated ischemic cardiomyopathy. Cytotherapy.

[29] Al-Saqi SH (2015). Defined serum-and xeno-free cryopreservation of mesenchymal stem cells. Cell and tissue banking.

[30] Wang C, Xiao R, Cao Y-L, Yin H-Y (2017). Evaluation of human platelet lysate and dimethyl sulfoxide as cryoprotectants for the cryopreservation of human adipose-derived stem cells. Biochemical and biophysical research communications.

[31] Karlsen TA, Brinchmann JE (2019). Expression of inflammatory cytokines in mesenchymal stromal cells is sensitive to culture conditions and simple cell manipulations. Experimental cell research.

[32] Ye JY (2010). Platelet-derived growth factor enhances platelet recovery in a murine model of radiation-induced thrombocytopenia and reduces apoptosis in megakaryocytes via its receptors and the PI3-k/Akt pathway. Haematologica.

[33] Bara, J. J., Richards, R. G., Alini, M. & Stoddart, M. J. J. S. C. Concise review: Bone marrow‐derived mesenchymal stem cells change phenotype following *in vitro* culture: implications for basic research and the clinic. **32**, 1713–1723 (2014). 10.1002/stem.1649 24449458

[34] Kehler J (2004). Oct4 is required for primordial germ cell survival. EMBO reports.

[35] Chambers I (2003). Functional expression cloning of Nanog, a pluripotency sustaining factor in embryonic stem cells. Cell.

[36] Tsai C-C, Su P-F, Huang Y-F, Yew T-L, Hung S-C (2012). Oct4 and Nanog directly regulate Dnmt1 to maintain self-renewal and undifferentiated state in mesenchymal stem cells. Molecular cell.

[37] Castiglia S (2014). Inactivated human platelet lysate with psoralen: a new perspective for mesenchymal stromal cell production in Good Manufacturing Practice conditions. Cytotherapy.

[38] Boiret N (2003). CD34+ CDw90 (Thy-1)+ subset colocated with mesenchymal progenitors in human normal bone marrow hematon units is enriched in colony-forming unit megakaryocytes and long-term culture-initiating cells. Experimental hematology.

[39] Bühring HJ (2009). Phenotypic characterization of distinct human bone marrow–derived MSC subsets. Annals of the New York Academy of Sciences.

[40] Zaky S, Ottonello A, Strada P, Cancedda R, Mastrogiacomo M (2008). Platelet lysate favours *in vitro* expansion of human bone marrow stromal cells for bone and cartilage engineering. Journal of tissue engineering and regenerative medicine.

[41] Lange C, Brunswig-Spickenheier B, Eissing L, Scheja L (2012). Platelet lysate suppresses the expression of lipocalin-type prostaglandin D2 synthase that positively controls adipogenic differentiation of human mesenchymal stromal cells. Experimental cell research.

[42] Torensma R (2012). The impact of cell source, culture methodology, culture location, and individual donors on gene expression profiles of bone marrow-derived and adipose-derived stromal cells. Stem cells and development.

[43] Lohmann M (2012). Donor age of human platelet lysate affects proliferation and differentiation of mesenchymal stem cells. PloS one.

[44] Pösel C (2012). Density gradient centrifugation compromises bone marrow mononuclear cell yield. PloS one.

[45] Horn, P., Bork, S. & Wagner, W. In Mesenchymal Stem Cell Assays and Applications 23–35 (Springer, 2011).

[46] Fennema EM, Renard AJ, Leusink A, van Blitterswijk CA, de Boer J (2009). The effect of bone marrow aspiration strategy on the yield and quality of human mesenchymal stem cells. Acta orthopaedica.

[47] Martin I, Muraglia A, Campanile G, Cancedda R, Quarto R (1997). Fibroblast growth factor-2 supports *ex vivo* expansion and maintenance of osteogenic precursors from human bone marrow. Endocrinology.

[48] Di Maggio N (2012). Fibroblast growth factor‐2 maintains a niche‐dependent population of self‐renewing highly potent non‐adherent mesenchymal progenitors through FGFR2c. Stem Cells.

[49] Tarte, K. *et al*. Clinical-grade production of human mesenchymal stromal cells: occurrence of aneuploidy without transformation. **115**, 1549–1553 (2010). 10.1182/blood-2009-05-219907 20032501

[50] Rothe, L. *et al*. Human osteoclasts and osteoclast-like cells synthesize and release high basal and inflammatory stimulated levels of the potent chemokine interleukin-8. **139**, 4353–4363 (1998). 10.1210/endo.139.10.6247 9751519

[51] Prystaz, K. *et al*. Distinct effects of IL-6 classic and trans-signaling in bone fracture healing. **188**, 474–490 (2018). 10.1016/j.ajpath.2017.10.011 29146294

[52] Azouna, N. B. *et al*. Phenotypical and functional characteristics of mesenchymal stem cells from bone marrow: comparison of culture using different media supplemented with human platelet lysate or fetal bovine serum. **3**, 6 (2012). 10.1186/scrt97 PMC3340550 22333342

[53] Crapnell, K. *et al*. Growth, differentiation capacity, and function of mesenchymal stem cells expanded in serum-free medium developed via combinatorial screening. **319**, 1409–1418 (2013). 10.1016/j.yexcr.2013.04.004 23597555

[54] Saldaña L (2019). Immunoregulatory potential of mesenchymal stem cells following activation by macrophage-derived soluble factors. Stem cell research & therapy.

[55] Grau-Vorster M (2019). Levels of IL-17F and IL-33 correlate with HLA-DR activation in clinical-grade human bone marrow–derived multipotent mesenchymal stromal cell expansion cultures. Cytotherapy.

[56] Grau-Vorster M, Laitinen A, Nystedt J, Vives J (2019). HLA-DR expression in clinical-grade bone marrow-derived multipotent mesenchymal stromal cells: a two-site study. Stem cell research & therapy.

[57] Sun YQ (2015). Insensitivity of human iPS cells‐derived mesenchymal stem cells to interferon‐γ‐induced HLA expression potentiates repair efficiency of hind limb ischemia in immune humanized NOD Scid gamma mice. Stem Cells.

[58] Fu X (2015). Comparison of immunological characteristics of mesenchymal stem cells derived from human embryonic stem cells and bone marrow. Tissue Engineering Part A.

[59] Bocelli‐Tyndall C (2010). Fibroblast growth factor 2 and platelet‐derived growth factor, but not platelet lysate, induce proliferation‐dependent, functional class II major histocompatibility complex antigen in human mesenchymal stem cells. Arthritis & Rheumatism.

[60] Gharibi, B. & Hughes, F. J. J. S. C. T. M. Effects of medium supplements on proliferation, differentiation potential, and *in vitro* expansion of mesenchymal stem cells. **1**, 771–782 (2012). 10.5966/sctm.2010-0031 PMC3659663 23197689

[61] Hemeda H, Kalz J, Walenda G, Lohmann M, Wagner WJC (2013). Heparin concentration is critical for cell culture with human platelet lysate..

[62] Fennema, E. M. *et al*. Ectopic bone formation by aggregated mesenchymal stem cells from bone marrow and adipose tissue: A comparative study. **12**, e150–e158 (2018). 10.1002/term.2453 28485099

[63] Gamblin, A.-L. *et al*. Bone tissue formation with human mesenchymal stem cells and biphasic calcium phosphate ceramics: the local implication of osteoclasts and macrophages. **35**, 9660–9667 (2014). 10.1016/j.biomaterials.2014.08.018 25176068

[64] Kitaori, T. *et al*. Stromal cell–derived factor 1/CXCR4 signaling is critical for the recruitment of mesenchymal stem cells to the fracture site during skeletal repair in a mouse model. **60**, 813–823 (2009). 10.1002/art.24330 19248097

[65] Zhu, H. *et al*. The role of the hyaluronan receptor CD44 in mesenchymal stem cell migration in the extracellular matrix. **24**, 928–935 (2006). 10.1634/stemcells.2005-0186 16306150

[66] Ponte, A. L. *et al*. The *in vitro* migration capacity of human bone marrow mesenchymal stem cells: comparison of chemokine and growth factor chemotactic activities. **25**, 1737–1745 (2007). 10.1634/stemcells.2007-0054 17395768

[67] Wagner W (2008). Replicative senescence of mesenchymal stem cells: a continuous and organized process. PloS one.

[68] Schallmoser K (2010). Replicative senescence-associated gene expression changes in mesenchymal stromal cells are similar under different culture conditions. Haematologica.

[69] Zhang Z (2017). CRISPR/Cas9 genome-editing system in human stem cells: current status and future prospects. Molecular Therapy-Nucleic Acids.

[70] Oliveira, L., Dos, A. S., Parreira, R., Pinto, M. & Resende, R. Enhancing the Therapeutic Potential of Mesenchymal Stem Cells with the CRISPR-Cas System. *Stem cell reviews* (2019). 10.1007/s12015-019-09897-0 31147819

[71] Bougioukli, S. *et al*. *Ex vivo* gene therapy using human bone marrow cells overexpressing BMP-2: “Next-day” gene therapy versus standard “two-step” approach. *Bone* (2019). 10.1016/j.bone.2019.08.005 PMC6813891 31398502

