# Supplementary material for: Impact of humanised isolation and culture conditions on stemness and osteogenic potential of bone marrow derived mesenchymal stromal cells
Source: Sci Rep. 2019 Nov 5;9:16031. doi: 10.1038/s41598-019-52442-9 (PMC6831606; doi:10.1038/s41598-019-52442-9)
Supplement: Supplementary file 1 — Supplementary Dataset 1 [file 41598_2019_52442_MOESM1_ESM.pdf]

Impact of humanised isolation and culture conditions on stemness and osteogenic potential of bone marrow derived mesenchymal stromal cells

Salwa Suliman[1*], Hassan R. W. Ali[1], Tommy A. Karlsen[2], Jerome Amiaud[3], Samih Mohamed-Ahmed[1], Pierre Layrolle[3], Daniela E. Costea[4,5,6], Jan E. Brinchmann[2,7], Kamal Mustafa[1*]

Supplementary Table 1. Real time qPCR primers

GENE (HUMAN)	ASSAY ID	AMPLICON LENGTH
GAPDH	Hs 02758991_g1	93
CD90/THY1	Hs 00174816_m1	60
NANOG	Hs 02387400_g1	109
Oct-4/POU5F1	Hs 00999632_g1	77
IL-1α	Hs 001714092_m1	69
IL-1β	Hs 01555410_m1	91
IL-6	Hs 00174131_m1	95
CXCL8/IL-8	Hs 00174103_m1	101
RUNX2	Hs 01047973_m1	86
COL1α1	Hs 00164004_m1	66
ALP	H s01029144_m1	79
BMP-2	Hs 00154192_m1	60
BSP/IBSP	Hs00913377_m1	87
OC/BGLAP	Hs 01587813_g1	166
PPARG	Hs 00234592_m1	77
CEBPA	Hs 00269972_s1	77
LPL	Hs 00173425_m1	103
PARG1/ARHGAP2	Hs 00191351_m1	69
CDKN2A	Hs 00923894_m1	115
PTN	Hs 01085691_m1	62
**GENE (MOUSE)**	**ASSAY ID**	**AMPLICON LENGTH**
GAPDH	Mm 99999915_g1	109
IL-1α	Mm00439620_m1	68
IL-1β	Mm 00434228_m1	90
IL-6	Mm 00446190_m1	78
IL-8/CXCL15	Mm 00441263_m1	66
RUNX2	Mm 00501583_m1	65
COL1α2	Mm 00801666_g1	89
ALP	Mm 00475834_m1	65
